# The Host Microbiota Contributes to Early Protection Against Lung Colonization by *Mycobacterium tuberculosis*

**DOI:** 10.3389/fimmu.2018.02656

**Published:** 2018-11-14

**Authors:** Alexia Dumas, Dan Corral, André Colom, Florence Levillain, Antonio Peixoto, Denis Hudrisier, Yannick Poquet, Olivier Neyrolles

**Affiliations:** Institut de Pharmacologie et de Biologie Structurale, IPBS, Université de Toulouse, CNRS, UPS, Toulouse, France

**Keywords:** microbiota, MAIT cells, macrophage, tuberculosis, IL-17

## Abstract

Tuberculosis (TB), caused by the airborne bacterial pathogen *Mycobacterium tuberculosis*, remains a major source of morbidity and mortality worldwide. So far, the study of host-pathogen interactions in TB has mostly focused on the physiology and virulence of the pathogen, as well as, on the various innate and adaptive immune compartments of the host. Microbial organisms endogenous to our body, the so-called microbiota, interact not only with invading pathogens, but also with our immune system. Yet, the impact of the microbiota on host defense against *M. tuberculosis* remains poorly understood. In order to address this question, we adapted a robust and reproducible mouse model of microbial dysbiosis based on a combination of wide-spectrum antibiotics. We found that microbiota dysbiosis resulted in an increased early colonization of the lungs by *M. tuberculosis* during the first week of infection, correlating with an altered diversity of the gut microbiota during this time period. At the cellular level, no significant difference in the recruitment of conventional myeloid cells, including macrophages, dendritic cells and neutrophils, to the lungs could be detected during the first week of infection between microbiota-competent and -deficient mice. At the molecular level, microbiota depletion did not impact the global production of pro-inflammatory cytokines, such as interferon (IFN)γ, tumor necrosis factor (TNF)α and interleukin (IL)-1β in the lungs. Strikingly, a reduced number of mucosal-associated invariant T (MAIT) cells, a population of innate-like lymphocytes whose development is known to depend on the host microbiota, was observed in the lungs of the antibiotics-treated animals after 1week of infection. These cells produced less IL-17A in antibiotics-treated mice. Notably, dysbiosis correction through the inoculation of a complex microbiota in antibiotics-treated animals reversed these phenotypes and improved the ability of MAIT cells to proliferate. Altogether, our results demonstrate that the host microbiota contributes to early protection of lung colonization by *M. tuberculosis*, possibly through sustaining the function(s) of MAIT cells. Our study calls for a better understanding of the impact of the microbiota on host-pathogen interactions in TB. Ultimately, this study may help to develop novel therapeutic approaches based on the use of beneficial microbes, or components thereof, to boost anti-mycobacterial immunity.

## Introduction

Tuberculosis (TB) remains a major public health issue, with over 10 million cases each year and 1.7 million deaths in 2016, according to the World Health Organization (WHO Annual report 2017). Understanding the host factors that modulate susceptibility or resistance to *Mycobacterium tuberculosis*, the etiological agent of TB, might help develop novel intervention strategies, including a more efficacious alternative to the Bacillus Calmette-Guérin (BCG) vaccine, as well as, host-directed therapies (1).

A key parameter controlling susceptibility to TB is the balance between the virulence of the infecting *M. tuberculosis* strain, and the immune status of the infected host. To date, the parameters involved in this balance mostly included the host genetic background, gender, age, nutrition status, and the occurrence of human immunodeficiency virus (HIV) co-infection or diabetes co-morbidity (2). The host microbiota, which represents the entirety of the microbial communities present on the skin and the mucosal surfaces of the body, might constitute an important but yet poorly explored additional factor influencing *M. tuberculosis* interaction with its host, and susceptibility to TB (3, 4).

The microbiota includes mostly bacteria, and profoundly influences human health and disease (5, 6). In mice, some evidence demonstrate its beneficial role, including resistance to infection by pathogenic microorganisms (7, 8). For example, segmented filamentous bacteria (SFB) present in the gut are sufficient to induce the accumulation of T helper (Th)17 cells in the lamina propria and to protect against infection by intestinal pathogens, such as *Citrobacter rodentium* (9). SFB were also reported to promote host resistance to pathogens at distal tissue sites, including the lung (10), which is now commonly referred to as the “gut-lung axis” (11, 12). In addition, the host microbiota is known to promote tolerance and immune homeostasis, such as through sustaining the development of FoxP3^+^ regulatory T cells (Tregs) in the colon (13, 14). Although the microbiota predominates in the gut, in which it has been mostly studied, it is present in all other mucosal surfaces of the body. The lung is no exception and it has become increasingly apparent over the past years that, although considered sterile for decades, the mammalian respiratory tract harbors a genuine microbiota as well (15–17). Yet, the exact role that these microbial communities play in susceptibility or resistance to respiratory diseases still remains largely enigmatic.

In TB, variations in the composition of the host intestinal and lung microbiota have been reported in various settings, including in patients and in animal models (18–20). However, from a functional viewpoint, whether the host microbiota contributes to resistance or susceptibility to TB, and how it does so, is still poorly understood. In particular, it is not known whether the microbiota modulates lung immunity in response to *M. tuberculosis* infection; a question that we have addressed in the present study.

To explore the role of the microbiota in physiological processes, including immunity, two mouse models are classically used, namely germ-Free (GF) mice, which are born and raised in sterile conditions, and mice treated with various cocktails of broad-spectrum antibiotics. Because the microbiota is involved in the education of the immune system, particularly during infancy, it is well-known that GF mice display abnormal immune functions, such as an immature and underdeveloped lymphoid system (21). This is not the case in antibiotics-treated mice, which are only transiently depleted of their microbiota and possess a mature immune system (22).

Here, we adapted an antibiotic treatment-based model of microbiota dysbiosis (23) in order to study the role of the microbiota on host susceptibility to *M. tuberculosis* infection and anti-mycobacterial immunity. We found that dysbiosis following antibiotic treatment strongly altered the diversity of the gut microbiota, but not that of the lung community, and increased early lung colonization by the TB bacillus. These phenotypes were reversed after microbial reconstitution of antibiotics-treated mice through fecal transplantation (FT). Strikingly, impaired protection against *M. tuberculosis* infection in antibiotics-treated mice correlated with a decreased number of lung mucosal associated invariant T (MAIT) cells, a population of innate-like lymphocytes associated with protection against pulmonary pathogens (24, 25) and whose development is known to be microbiota-dependent (26). Moreover, MAIT cells in antibiotics-treated mice produced less interleukin (IL)-17A, and MAIT cells from FT mice proliferated more than those from antibiotics-treated mice following infection.

Altogether, our results reveal that the presence of a healthy microbiota promotes host resistance to early colonization by *M. tuberculosis*, possibly through sustaining the function(s) of MAIT lymphocytes.

## Materials and methods

### Animal models of dysbiosis, microbiota restoration and *M. tuberculosis* infection

Six-to-eight week-old female C57BL/6 mice were purchased from Charles River Laboratories. Mice were given a combination of broad-spectrum antibiotics *ad libitum* in drinking water. Mice were given ampicillin (1 g/L, Sigma Aldrich), vancomycin (500 mg/L, Alfa Aesar) and neomycin sulfate (1 g/L, Fisher) during 2 weeks, and this treatment was complemented with metronidazole during the third (0.5 g/L, Alfa Aesar) and fourth (1 g/L) weeks. Antibiotics-containing water was changed twice a week, and treatment was stopped 2 days before *M. tuberculosis* infection or fecal transplantation. Microbial depletion was confirmed by checking for the absence of live microorganisms in the feces using the colorimetric Releasat biological indicator (Mesa Labs) at 37°C, in either aerobic or anaerobic conditions. Following microbial depletion, mice were maintained in a sterile isolator during all the experiment. Recolonization with a complex microbiota was performed through a single gavage with the intestinal content from two non-treated mice. Repopulation occurred after 1 week, as assessed using the colorimetric Releasat biological indicator. Control mice received normal water during all experiment.

*M. tuberculosis* (H37Rv) was grown in 7H9 liquid medium (Difco) supplemented with ADC (Middlebrook) and tyloxapol 0.05%. For infection, mice were anesthetized using isoflurane and infected intranasally with 10^3^ colony-forming units (CFUs) of *M. tuberculosis*.

### Preparation of lungs homogenates for microbiological, molecular, and immunological analyses

Mice were sacrificed, lungs were harvested aseptically, homogenized using a gentleMACS dissociator (C Tubes, Miltenyi), and incubated with DNAse I (0.1 mg/mL, Roche) and collagenase D (2 mg/mL, Roche) during 30 min at 37°C under 5% CO_2_. *M. tuberculosis* bacterial load was determined by plating serial dilutions of the lung homogenates onto 7H11 solid medium (Difco) supplemented with OADC (Middlebrook). The plates were incubated at 37°C for 3 weeks before bacterial CFUs scoring. Lungs homogenates were filtered on 40 μm cell strainers and centrifuged at 329 × *g* during 5 min. Supernatants were conserved for cytokine content analysis. A part of the cellular pellet was conserved in TRIzol reagent for cellular RNA analysis. In the remaining fraction, red blood cells were lysed in 150 mM NH_4_Cl, 10 mM KHCO_3_, 0.1 mM EDTA (pH 7.2) for immunological staining. For microbial composition analysis, lungs were harvested aseptically, homogenized using a gentleMACS dissociator (M Tubes, Miltenyi), and filtered on 40 μm cell strainers.

### *In vitro* stimulation, antibody staining, and flow cytometry analysis

Single-cell suspensions from lungs homogenates were prepared as described above. In case of intracellular staining of lymphocytes, cytokine production was stimulated in RPMI (Difco) supplemented with phorbol myristate acetate (PMA) and ionomycin (50 ng/mL and 1 μg/mL, respectively, Sigma Aldrich), and blocked with brefeldin A and monensin (5 μg/mL, Becton Dickinson) for 4 h at 37°C under 5% CO_2_. In case of intracellular staining of myeloid cells, cytokine production was stimulated in RPMI (Difco) supplemented with lipopolysaccharide (LPS) (1 μg/mL, Sigma Aldrich), and blocked with brefeldin A (5 μg/mL, Becton Dickinson) for 4 h at 37°C under 5% CO_2_. Cells were stained with Zombie Aqua Viability for dead cells exclusion and non-specific binding to Fc receptors was blocked by incubating the cells with anti-CD16/32 antibodies (BioLegend). For MAIT cell staining, mouse MR1-5-OP-RU tetramer labeled with PE and MR1-Ac-6-FP tetramer labeled with APC were incubated for 45 min at room temperature in the dark. The MR1 tetramer was obtained from the NIH Tetramer Core Facility. For extracellular staining, cells were incubated with a panel of surface antibodies for 20 min at 4°C in the dark. For intracellular staining, cells were fixed and permeabilized with Fixation and Permeabilization Reagents (eBioscience) and subsequently stained with the antibodies of interest for 40 min at room temperature in the dark. All antibodies used are referenced in Supplementary Table 1. Cell staining was analyzed using LSR II or LSR Fortessa flow cytometers (BD) and FlowJo software version V10. Cells were first gated on singlets (FSC-H vs. FSC-W and SSC-H vs. SSC-W) and live cells before further analyses. An average of 10–25,000 alveolar macrophages were sorted using a FACSAria Fusion cytometer. Sorted cells were incubated in TRIzol reagent to isolate RNA.

### Bacterial DNA and cellular RNA isolation, and real Time-qPCR

DNA from fecal samples and lungs homogenates were extracted according to manufacturer's instructions (QIAamp Fast DNA Stool Mini Kit, Qiagen). DNA was extracted from the fecal samples, using lab tissue-specific technique. The 16S rDNA present in the samples was measured by Real Time-qPCR (RT-qPCR) in triplicate and normalized using a plasmid-based standard scale. The amount of bacterial DNA was assessed using the “Universal 16S Real Time qPCR” workflow established by Vaiomer (Vaiomer SAS, Labège, France). In the case of detection of bacterial phylums from lungs and feces DNA, RT-qPCR was performed using phylum-specific primers [Supplementary Table 2, (8, 27)] using TB green Premix Ex Taq (Takara), according to the manufacturer's protocol. All RT-qPCR reactions were carried out using a 7,500 RT-PCR System, and data were analyzed using the 7,500 Software version v2.3 (Applied Biosystems). Values were normalized using the universal 16S rRNA gene, and expressed as a fold change in experimental samples (antibiotics-treated mice or reconstituted mice) relative to control samples (non-treated mice). RNA from lungs homogenates and sorted cells were extracted using TRIzol reagent (Ambion) and RNeasy spin columns according to manufacturer's instructions (RNeasy kit, Qiagen). RNA were reverse transcribed into cDNA using M-MLV Reverse transcriptase (Invitrogen). RT-qPCR was performed using gene-targeted primers (Supplementary Table 2) as described above. Values were normalized using the *Hprt* housekeeping gene, and expressed as a fold change in experimental samples (antibiotics-treated mice) relative to control samples (non-treated mice).

### Cytokine quantification

Cytokines present in the supernatants from lung homogenates were quantified using a customized LEGENDplex™ Mouse Inflammation Panel and a LSRII flow cytometer, using manufacturer's instructions (BioLegend). CCL20 was detected by ELISA (Invitrogen), according to the manufacturer's instructions.

### Statistical analysis

Statistical analyses were performed using GraphPad Prism 7 software. An Agostino and Pearson normality test was performed to determine whether samples followed a normal distribution. Data following a normal distribution are represented as mean ± SD; data following a non-normal distribution were represented as median with interquartile range. Unpaired *t*-test (for normal data) or Mann-Whitney (for non-normal data) were performed when two samples were compared; ANOVA (for normal data) or Kruskal-Wallis (for non-normal data) tests were performed when more than two samples were compared. For all analyses, ^*^ indicates *P* < 0.05, ^**^ indicates *P* < 0.01, ^***^ indicates *P* < 0.001, and ^****^ indicates *P* < 0.0001. Data obtained from FT mice were included in the figures only when statistical difference between antibiotics-treated and control mice were detected.

## Results

### Microbiota dysbiosis increases early lung colonization by *M. tuberculosis*

**Figure 1:**
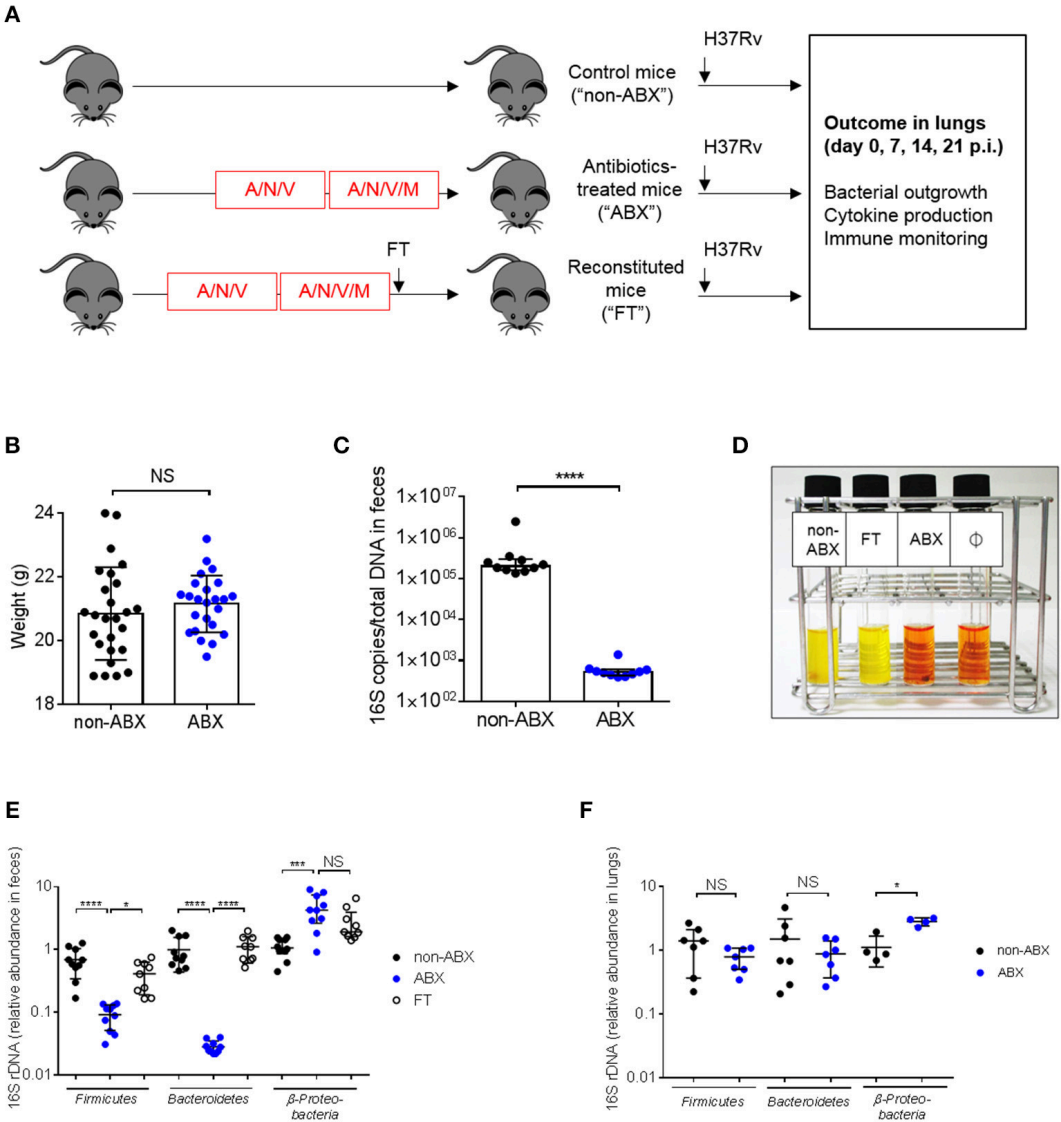
Mouse model to study the impact of microbiota dysbiosis during *M. tuberculosis* infection. **(A)** Experimental design. Six-to-eight weeks old C57BL/6 female mice were treated for 4 weeks with a cocktail of broad-spectrum antibiotics (ampicillin, A, neomycin sulfate, N, vancomycin, V, and metronidazole, M, which was added during the last 2 weeks) in drinking water (ABX) or were given water without antibiotics (non-ABX). Microbiota reconstitution was realized through fecal transplantation (FT) after a single oral gavage with the intestinal content from 2 control mice and occurred during 1 week. The three groups of mice received an intranasal challenge of *M. tuberculosis* H37Rv (10^3^ CFUs/mouse). Bacterial outgrowth, cytokine production and immune monitoring were determined 0, 7, 14, or 21 days post-infection (p.i.) in the lungs. **(B)** Mouse weight in the ABX vs. non-ABX groups at the time of *M. tuberculosis* infection. Data from 4 independent experiments (*n* = 4–14 mice/group/experiment) were pooled and the graph represent mean ± SD of the pooled data. Data were analyzed using the unpaired Student's *t*-test. **(C)** The amount of bacterial rDNA was assessed using the “Universal 16S RT-qPCR” kit, calculated as 16S rDNA gene copies per total DNA extracted from feces of non-ABX and ABX groups before infection. **(D)** Colorimetric evaluation of the presence of microbial flora in the feces of the different groups of animals before infection; Ø indicates negative control. Yellow color indicates the presence of live microorganisms. Image depicted in **(D)** is representative of 3 independent experiments. Fecal **(E)** and lung **(F)** microbiota were detected by RT-qPCR using phylum-specific primers. Relative Ct value compared to universal 16S rRNA gene Ct value (ΔCt) and the mean of ΔCt values in the control group (ΔΔCt). **(E, F)** data from 2–3 independent experiments (*n* = 2–4 mice/group/experiment) were pooled. The graph **(E)** show mean ± SD (*Firmicutes* and *Bacteroidetes*) and median with interquartile range (*Proteobacteria*) of the pooled data. Data were analyzed using ANOVA (*Firmicutes* and *Bacteroidetes*) and the Kruskal-Wallis test (*Proteobacteria*). The graph **(F)** show median with interquartile range of the pooled data. Data were analyzed using the Mann-Whitney test. NS, not significant; *p < 0.05; ***p < 0.001; ****p < 0.0001.

In order to assess whether the microbiota is involved in the control of *M. tuberculosis* infection, we adapted a model of microbiota dysbiosis consisting in the administration of a cocktail of broad-spectrum antibiotics composed of ampicillin, neomycin sulfate, metronidazole, and vancomycin over the course of 4 weeks. This procedure has previously been shown to deplete all detectable commensals and bacterial products (23). We observed that adding metronidazole at the start of antibiotic treatment resulted in animal morbidity and mortality, presumably due to dehydration caused by a reluctance of the mice to drink metronidazole-containing water (28). Thus, the protocol was modified and metronidazole was added only during the last 2 weeks of antibiotic treatment (Figure 1A). In this way, mice did not suffer from dehydration and did not lose weight during the course of treatment (Figure 1B). Microbial depletion before *M. tuberculosis* infection was confirmed by quantification of total 16S rDNA in feces (Figure 1C) and by inoculation of fecal samples in reporter liquid medium incubated under aerobic (Figure 1D) or anaerobic (data not shown) conditions. Bacterial diversity was analyzed at the phylum level in control and antibiotics-treated (ABX) mice. *Bacteroidetes* and *Firmicute*s, the major phylums present in the gut microbiota (29) were strongly reduced, while *Beta-proteobacteria* were increased in the gut of ABX mice, compared to control animals (Figure 1E). An increase in *Proteobacteria* is known to be associated with gut inflammation and a bad prognostic during intestinal diseases (30, 31). We also verified if the lung community could be altered by antibiotics treatment. Colorimetric analyses did not reveal the presence of viable bacteria in the broncho-alveolar lavages (BALs) of normal, specific pathogen-free (SPF) mice (data not shown). A similar trend was observed in the lungs for the major phyla (17, 32), although statistical significance was reached only for Proteobacteria (Figure 1F).

In order to confirm the direct contribution of the host microbiota to the observed phenotypes, we generated groups of ABX mice reconstituted with the intestinal content from untreated control mice through fecal transplantation (FT). Some features of normal mice were restored in ABX mice after FT (Figures 1D,E). In particular, the diversity of the major phylums (e.g., *Bacteroidetes* and *Firmicutes*) was similar between control and FT mice (Figure 1E).

We next verified that the antibiotic treatment did not alter the level and functionality of several lung immune cell populations at the steady state (Supplementary Figure 1). The total numbers of macrophages, dendritic cells, neutrophils, natural killer (NK), CD4 T lymphocytes, and B cells were quantified by flow cytometry. We found no difference in the number of these cells (Supplementary Figure 1A), and in the ability of macrophages and CD4 T cells to produce cytokines (Supplementary Figure 1B) between control and ABX mice.

**Figure 2:**
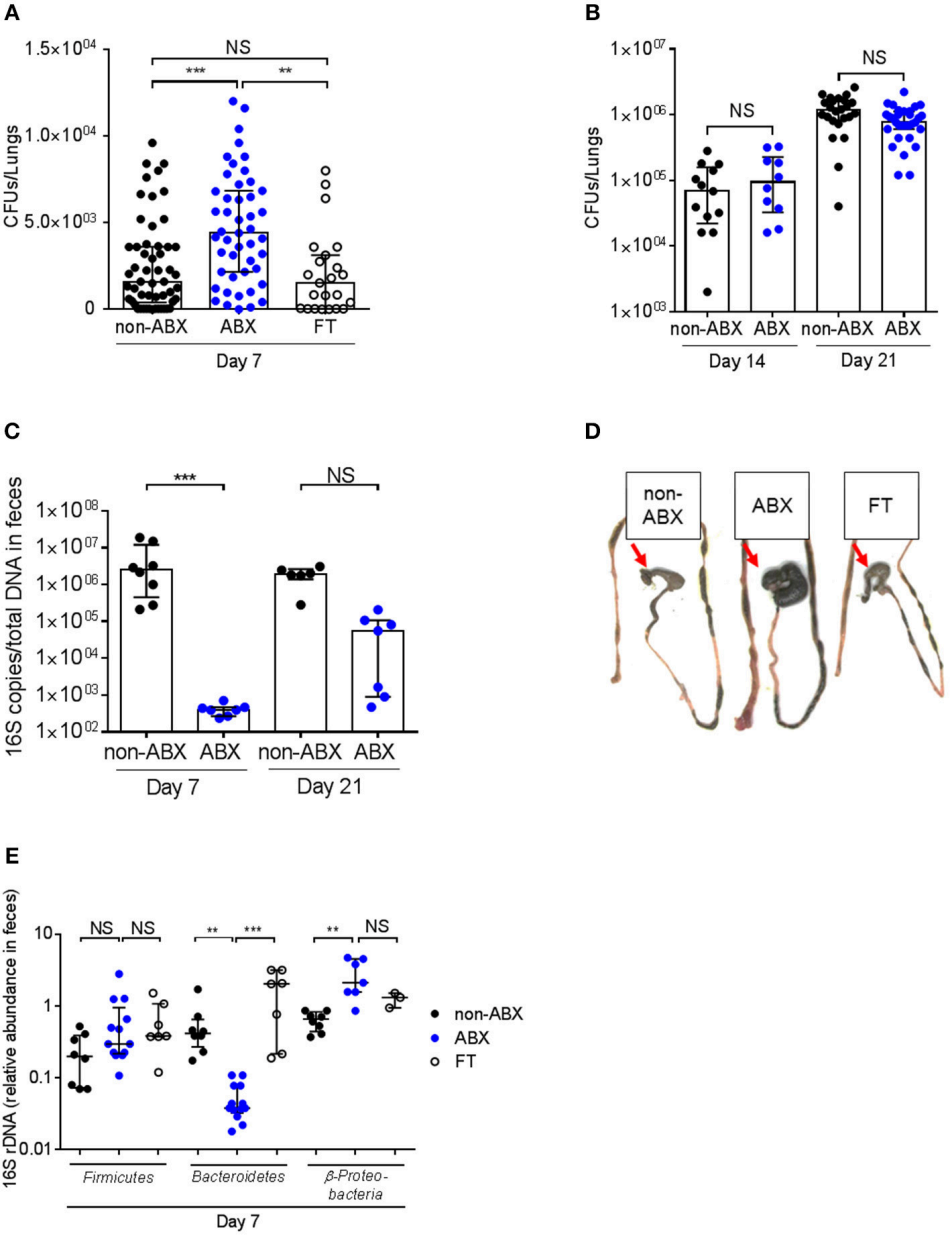
The host microbiota protects against early lung colonization by *M. tuberculosis*. **(A) ***M. tuberculosis* bacterial load (CFUs) was measured in the lungs of control (non-ABX), ABX and FT mice 7 days p.i. **(B) ***M. tuberculosis* bacterial load was measured in the lungs of control (non-ABX) and ABX mice 14 or 21 days p.i., Data from 3–6 **(A)** or 2 **(B)** independent experiments (*n* = 4–8 mice/group/experiment) were pooled and the graphs show median with interquartile range of the pooled data. Data were analyzed using the Kruskal-Wallis test. **(C)** The amount of bacterial rDNA was assessed using the “Universal 16S RT-qPCR” kit, calculated as 16S rDNA gene copies per total DNA extracted from feces of non-ABX, ABX, and FT groups 7 days p.i. **(D)** Morphology of the caecum in the non-ABX, ABX, and FT groups 7 days p.i., Red arrow indicates the caecum. Image depicted in **(D)** is representative of 3 independent experiments. **(E)** Fecal microbiota from non-ABX, ABX and FT groups was analyzed by RT-qPCR using phylum-specific primers 7 days p.i., Data were analyzed as in Figure 1. **(C,E)** Data from 1–4 independent experiments (*n* = 2–5 mice/group/experiment) were pooled and the graphs show median with interquartile range of the pooled data. Data were analyzed using the Kruskal-Wallis test. NS, not significant; **p < 0.01; ***p < 0.001.

Control (non-ABX) and ABX mice were infected intranasally with *M. tuberculosis* H37Rv 2 days after cessation of antibiotics treatment, and the lungs of the infected animals were collected 7, 14, and 21 days after infection, homogenized and plated onto agar medium for colony-forming unit (CFUs) scoring. Compared to control mice, ABX mice contained about twice as many bacteria in their lungs at day 7 post-infection (p.i., Figure 2A). This phenotype was only transient since there was no difference in the lung bacterial load between control and ABX mice after 14 or 21 days p.i. (Figure 2B); similarly no differences in bacterial loads were observed in the spleen of control vs. ABX animals 21 days p.i. (data not shown). This phenotype correlated with a reduced amount of total gut microbial rDNA in ABX mice 7 days p.i, while this difference was abrogated 21 days p.i. (Figure 2C), indicating that ABX mice were recolonized following cessation of the antibiotic treatment. Yet, microbial recolonization over time in ABX mice was only partial, which correlated with the presence of an enlarged caecum in ABX mice, as previously observed in GF mice (21) (Figure 2D). The relative abundance of *Firmicutes* was no more different in infected ABX and control mice 7 days p.i., however the relative abundance of *Bacteroidetes* and *Proteobacteria* was still altered in ABX animals at this time point (Figure 2E), which likely reflected ongoing recolonization.

Reintroduction of a complex microbiota (FT) restored all the examined phenotypes, i.e., prevented the increased lung colonization by *M. tuberculosis* 7 days p.i. (Figure 2A), caecum enlargement (Figure 2C), and restored, at least partially, microbial diversity (Figure 2E).

Altogether, these data demonstrate that the host microbiota contributes to early resistance against lung colonization by *M. tuberculosis*.

### Microbiota dysbiosis correlates with impaired functions of mait cells during early lung colonization by *M. tuberculosis*

In order to understand the increased susceptibility of ABX mice to *M. tuberculosis*, we next quantified the early expression and production of several key inflammatory factors known to be involved in immunity to TB (33). Seven days after *M. tuberculosis* infection, we found no difference in the mRNA expression and/or protein production of TNFα, IL-1β, IL-6, transforming growth factor (TGF)β, interferon (IFN)γ, IL-17A and IL-12 (p70) in the lungs of ABX vs. control mice (Supplementary Figure 2). We also quantified the expression of cathelicidin-related antimicrobial peptide (CRAMP), whose production is known to be stimulated by the microbiota (34), and found no difference between ABX and control mice (Supplementary Figure 2A).

**Figure 3:**
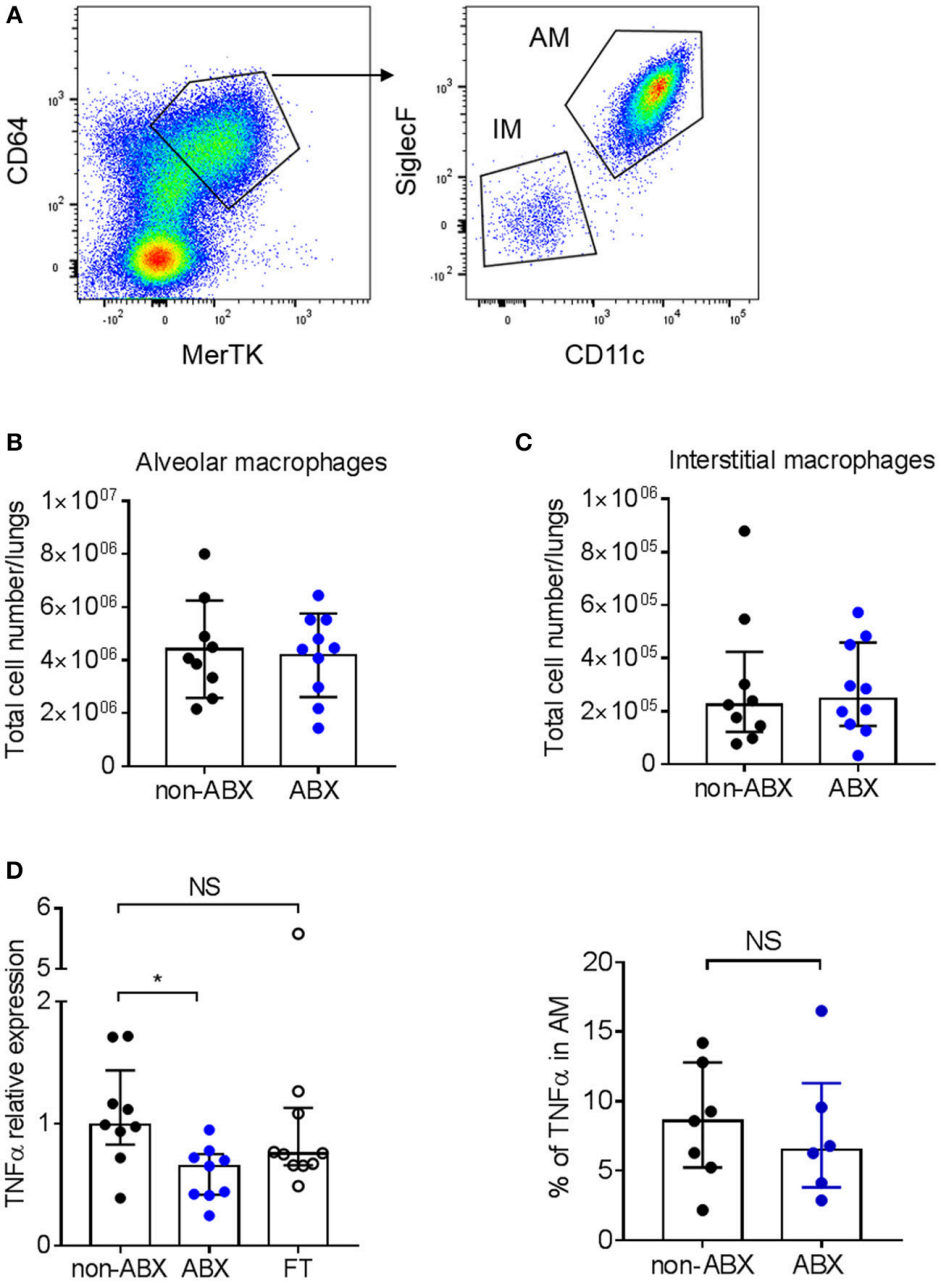
Microbiota dysbiosis modify TNFα mRNA expression but not protein production by alveolar macrophages. **(A)** Gating strategy to analyze alveolar (MerTK^+^CD64^+^SiglecF^+^CD11c^+^) and interstitial (MerTK^+^CD64^+^SiglecF^−^CD11c^−^) macrophages by flow cytometry. The total number of **(B)** alveolar and **(C)** interstitial macrophages was quantified by flow-cytometry in lung homogenates from *M. tuberculosis*-infected ABX vs. non-ABX mice 7 days p.i., Data from 2 independent experiments (*n* = 4–5 mice/group/experiment) were pooled and the graphs show mean ± SD **(B)** or median with interquartile range **(C)**. Data were analyzed using the Student's *t*-test **(B)** or the Mann-Whitney test **(C)**. **(D)** Left panel, MerTK^+^CD64^+^SiglecF^+^CD11c^+^ alveolar macrophages were sorted and the expression of TNFα was measured by RT-qPCR in cells from non-ABX, ABX, or FT mice 7 days p.i., Gene expression represents relative Ct value compared to *Hprt* Ct value (ΔCt) and the mean of ΔCt values in the control group (ΔΔCt). Right panel, cytometry analysis of the percentage of TNFα-producing MerTK^+^CD64^+^SiglecF^+^CD11c^+^ alveolar macrophages following *in vitro* stimulation by LPS. Data from 1–2 independent experiments (*n* = 4–5 mice/group/experiment) were pooled and the graphs show median with interquartile range of the pooled data and were analyzed using the Mann-Whitney test *p < 0.05.

We next analyzed the early accumulation of innate myeloid and lymphoid cell populations in the lungs of infected ABX and control mice by flow cytometry. We found no difference in the number of macrophages, dendritic cells, neutrophils and natural killer (NK) cells between the two groups of animals 7 days p.i. (Supplementary Figure 3). Macrophages are known to be the main host cell target for *M. tuberculosis*, and are able to control mycobacterial proliferation upon activation by inflammatory cytokines, including TNFα (33). In particular, alveolar macrophages (AMs) were recently reported to sustain mycobacterial proliferation while interstitial macrophages (IMs) are more bactericidal (35). Here we found no difference in the number of AMs and IMs between ABX and control mice 7 days p.i. (Figures 3A–C); however after sorting the AM population, we found that these cells expressed less TNFα mRNA in ABX mice, as compared to in control animals (Figure 3D, left panel). Yet, the production of TNFα by AMs in ABX and control mice was similar at the protein level, as assessed by flow cytometry (Figure 3D, right panel); similar results were obtained in IMs (data not shown).

**Figure 4:**
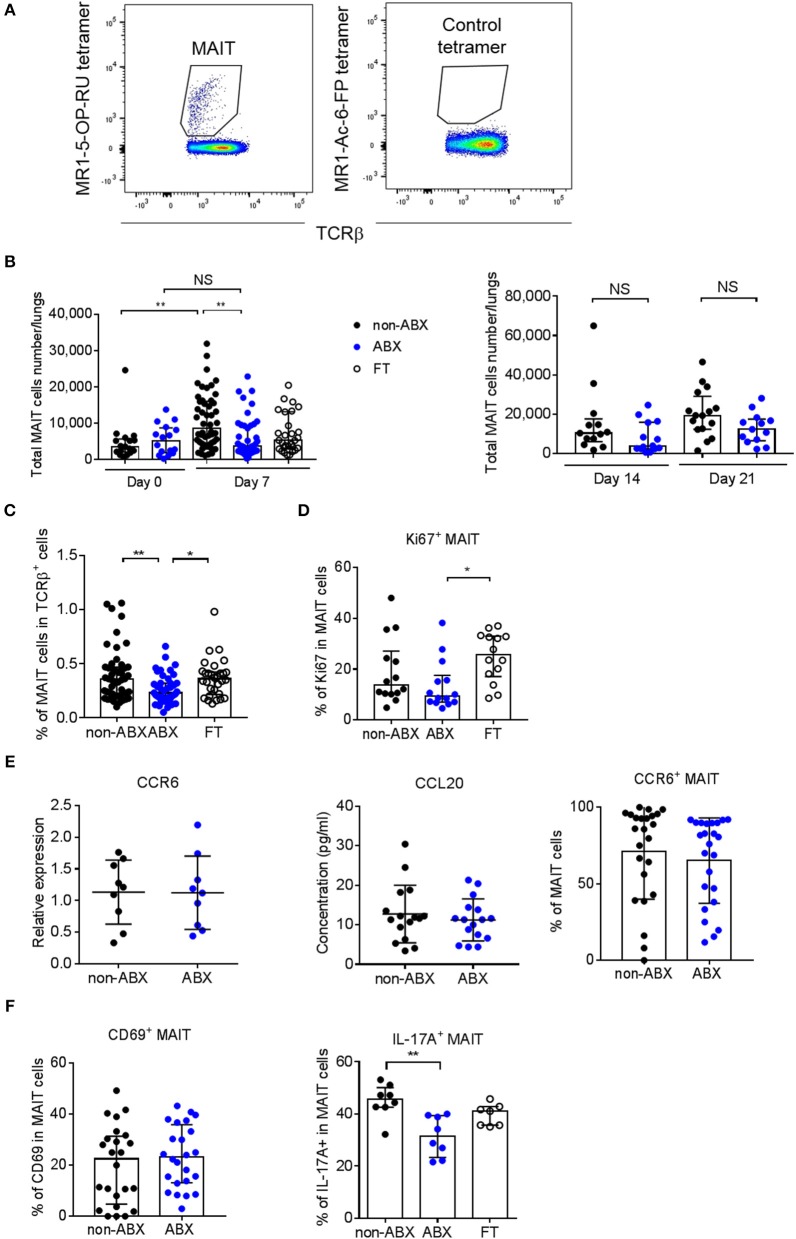
MAIT cell accumulation in the lungs and IL-17A-production are decreased early after *M. tuberculosis* infection in microbiota-altered mice. **(A)** Gating strategy to analyze MAIT cells (MR1-5-OP-RU tetramer^+^TCRβ^+^) by flow cytometry. A MR1–Ac-6-FP tetramer^+^TCRβ^+^ staining was used as a control. **(B,C)** Total number **(B)** and percentage **(C)** of MAIT cells in the lungs of control (non-ABX) ABX, and FT mice 0 and 7 (**B**, left panel), 14 and 21 (**B**, right panel), and 7 **(C)** days p.i., Data from 2–6 independent experiments (*n* = 4–8 mice/group/experiment) were pooled and the graphs show median with interquartile range of the pooled data. Data were analyzed using the Kruskal-Wallis test. **(D)** Intracellular analysis of Ki67 expression in MAIT cells from non-ABX, ABX, and FT mice 7 days p.i., by flow cytometry. Data from 2 independent experiments (*n* = 4–8 mice/group/experiment) were pooled and the graphs show the median with interquartile range of the pooled data. Data were analyzed using the Kruskal-Wallis test. **(E)** The expression of CCR6 (left) in whole lung homogenates or in MAIT cells (right), and the production of CCL20 in lung supernatant (middle), were measured by RT-qPCR (left), flow cytometry (right) and ELISA (middle) in ABX and control (non-ABX) mice 7 days p.i., RT-qPCR data were analyzed as in Supplementary Figure 2. Data from 3 (left) or 4 (middle and right) independent experiments (*n* = 3–7 mice/group/experiment) were pooled and the graphs show mean ± SD of the pooled data. Data were analyzed using the Student's *t*-test. **(F)** Cytometry analysis of percentage of activated (MR1-5-OP-RU tetramer^+^TCRβ^+^CD69^+^) MAIT cells (left), and IL-17A-producing (MR1-5-OP-RU tetramer^+^TCRβ^+^IL-17A^+^) MAIT cells (right) in lungs of control (non-ABX), ABX and FT mice 7 days p.i., Data from 4 (left) or 1 representative of 3 (right) independent experiments (*n* = 5–8 mice/group/experiment) were pooled and the graphs show mean ± SD of the pooled data. (left) or median with interquartile range (right) of the pooled data. Data were analyzed using the unpaired Student's *t*-test (left) or the Kruskal-Wallis test (right). NS, not significant; *p < 0.05; **p < 0.01.

Since the microbiota is known to play a part in the effector functions of innate-like lymphocytes (36), we next explored the accumulation of NKT cells, γ/δ T cells and MAIT cells in the lungs of ABX and control mice during *M. tuberculosis* infection. The total number and percentage of NKT (NK1.1+CD3+) and γ/δ T (TCRγ/δ+CD3+) lymphocytes were similar in the lungs of infected ABX and control mice (Supplementary Figure 4). Strikingly, the only difference we observed between the two groups of mice was related to the accumulation of mucosal-associated invariant T (MAIT) cells, characterized by their MR1-5-OP-RU tetramer+TCRβ+ phenotype (Figure 4A), with a marked decrease in the total number and percentage of these cells in ABX mice, compared to in control mice 7 days p.i. (Figures 4B,C). This phenotype was reversed in FT mice (Figures 4B,C) and was no longer observed at later time points p.i. (Figure 4B), in line with our findings for lung bacterial loads (Figures 2A,B). Altogether, these data suggest that the host microbiota promotes early resistance to *M. tuberculosis* infection through sustaining the accumulation of MAIT cells in the lungs.

We next sought to assess whether the decreased number of MAIT cells observed in the lungs of ABX mice reflected a diminished recruitment and/or proliferation of these cells following *M. tuberculosis* infection. We found that MAIT cells from ABX mice expressed less Ki67, an intracellular marker of cell cycling and proliferation, than their control or FT counterparts (Figure 4D). The tissue-homing chemokine receptor CCR6 and the chemokine CCL20 (referred to as the CCR6/CCL20 axis) are known to be involved in the recruitment of leukocytes, including T lymphocytes, from the intestine and other organs to the lungs (37, 38), and were found to be altered during microbiota dysbiosis in response to pulmonary infection (39). Because MAIT cells express CCR6 (40), we next determined if the CCR6/CCL20 axis was perturbed in ABX mice. We found no difference in global expression of the CCR6 transcript and production of the CCL20 protein in the lungs between *M. tuberculosis*-infected ABX and control mice 7 days p.i. (Figure 4E). Flow cytometry analysis revealed no difference in CCR6 expression in MAIT cells between the two groups of mice (Figure 4E). These data indicate that if microbiota dysbiosis impairs the early recruitment of MAIT cells to the lungs following *M. tuberculosis* infection, this does not rely on the CCR6/CCL20 axis. Moreover, they reveal that the proliferation of MAIT cells is reduced in the lungs of ABX mice.

Finally, we sought to evaluate whether the activation and effector functions of MAIT cells could be altered in the lungs of *M. tuberculosis*-infected ABX mice. Activated MAIT cells are known to express CD69 (25) and to produce various cytokines, including IL-17A (24, 41). In the mouse, lung MAIT cells mostly produce IL-17A (41). We found that although CD69 expression by MAIT cells was similar in ABX and control mice, MAIT cells from ABX animals produced less IL-17A, which was partially restored in FT mice 7 days p.i. (Figure 4F). The number of, and Ki67 expression by MAIT cells in the spleen did not differ between ABX and control animals. Altogether, our data reveal that a healthy microbiota is required for pulmonary MAIT cell functions, which correlates with an improved early control of *M. tuberculosis* growth in the lungs.

## Discussion

Our results demonstrate that the microbiota contributes to early host defense against lung colonization by *M. tuberculosis*, possibly *via* effector functions MAIT lymphocytes. Several previous studies reported that *M. tuberculosis* infection results in a perturbation of the composition of the host microbiota in humans, as well as, in experimentally infected animals (18, w^20^, ^42^). However, the functional impact of the microbiota on host susceptibility or resistance to TB remains to be fully understood.

In a previous study using a different ABX model, Khan and colleagues reported that antibiotics-treated mice were more susceptible to *M. tuberculosis*, with a higher bacillary load in the lungs 21 days after infection, compared to control mice (43). This correlated with a decrease in IFNγ- and TNFα-producing CD4 T lymphocytes and an increase in FoxP3^+^ Tregs in the spleen of the infected ABX animals, compared to controls. At first glance, these results would appear to contradict our data since we did not observe any difference in lung bacterial load in ABX vs. control animals at 14 or 21 days p.i., However, in our study antibiotic treatment was stopped 2 days before *M. tuberculosis* infection, while in the study by Khan and colleagues antibiotics were maintained in the drinking water throughout the infection period. It is likely that in our experiments, animals became progressively recolonized following recovery of the endogenous microflora in an uncontrolled manner, and this may explain why we only observed an increased susceptibility of ABX mice at early time-points during infection. Thus, our study and that by Khan et al. do not appear to be contradictory but rather complementary, illustrating the overall protective function of the host microbiota against *M. tuberculosis* infection at early vs. late time points post-exposure. Altogether, these two studies might reveal a previously underappreciated influence of the gut microbiota on host resistance to the TB bacillus through the so-called “gut-lung axis” (44), as observed in other lung infections (11, 45). Another, yet non-mutually exclusive possibility, is that local commensals of the airways (15, 16), which are probably also affected by antibiotics treatment (46), might influence host resistance to TB, which will need further investigation (47). A limitation in our study is that the composition of the microbiota in ABX mice was not monitored over time and that animals were infected only 2 days after cessation of the antibiotics treatment. The microbial composition in ABX mice in the weeks following cessation of antibiotics treatment is unlikely to be similar to that of control animals, and monitoring the microbial composition over time following cessation of antibiotics treatment would be an important study to perform. Similarly, assessing the impact of various microbiota, in particular of a “healthy microbiota,” on susceptibility to TB is an important issue that is not addressed here and would require developing different animal models.

We found that unconventional T cells, namely MAIT cells, might be involved in microbiota-mediated early control of *M. tuberculosis* infection, e.g., through the production of IL-17A. MAIT cells form a population of innate-like lymphocytes expressing a semi-invariant T cell antigen receptor (TCR), activated by the MHCI–related molecule MR1 presenting microbial-derived metabolites (48, 49). MAIT cells preferentially reside in mucosal tissues, such as the lungs and the gut, and their development and maturation depend on the presence of the host microbiota (26, 50). This explains why the MAIT cell compartment is disturbed in several inflammatory disorders in which the gut microbiota is altered, such as inflammatory bowel disease and obesity (51, 52). MAIT cells were found to be involved in host protection from a variety of pathogens, including respiratory pathogens such as *Klebsiella pneumoniae*, and *Francisella tularensis* (24, 53). Reminiscent of our study, mice lacking MAIT cells also display an increased early susceptibility to mycobacterial infections (25, 26, 54). In humans, TB patients display a functionally impaired MAIT cell compartment (55, 56). In our study, we found that MAIT cells tend to proliferate less in ABX mice, which may rely on a reduced availability of MAIT ligands in this setting (49, 57, 58). Although we cannot exclude that the recruitment of these cells to the lung is also diminished in ABX mice, we provide evidence that the reduced accumulation of MAIT cells in the lungs of ABX mice is independent of the CCR6/CCL20 axis. Yet, MAIT cells express other receptors involved in cell migration, namely CCR5, CCR9, CXCR4, and CXCR6, at least in humans (59). The role played by these receptors, and possibly others, in microbiota-mediated recruitment of MAIT cells to the lungs during *M. tuberculosis* infection, will need to be investigated. We found that MAIT cells produce less IL-17A in ABX mice. The role of IL-17 in TB is still unclear, possibly depending on the cell type producing this cytokine and the time point at which it is produced during infection (33). Whether the host microbiota influences early control of *M. tuberculosis* through the modulation of the production of IL-17, and possibly other cytokines, and how it does so, will need to be addressed. In the mouse, MAIT cells mostly produce IL-17A (41) but their ability to control *M. tuberculosis* in infected macrophages does not seem to depend on this cytokine (54), suggesting that the possible protective effect of IL-17A we observed is not direct. The development of MAIT cells is known to depend on the microbiota, particularly on microbes synthesizing riboflavin (26, 60). Addressing the role of specific microbiota species in the MAIT-mediated early protection against *M. tuberculosis* infection would require recolonizing ABX mice with riboflavin-producers (e.g., *Escherichia coli*) and -non producers (e.g., *Enterococcus faecalis*), which will be of interest for future studies.

In conclusion, we show here that the host microbiota contributes to early resistance to *M. tuberculosis* infection in mice, possibly through stimulating the accumulation and effector functions of MAIT cells in the lungs. Whether these findings apply to humans will need further investigation. A significant fraction of those individuals in close contact to TB cases are known to remain tuberculin skin test (TST)-negative, and are thus thought to represent “innate controllers” (61). It is tempting to speculate that in addition to other factors, the host microbiota might contribute to the ability to control *M. tuberculosis* infection in an innate fashion, *via* MAIT cells and possibly other innate cells (62).

## Ethics statement

Animal care and experimentation were performed following the French regulation. All procedures were approved by the Ministry of Higher Education and Research (Agreement APAFIS 6497).

## Author contributions

AD, DC, DH, YP, and ON designed research. AD, DC, FL, AC, and AP performed research. AD, DC, FL, AP, DH, YP, and ON analyzed data. AD and ON wrote the paper with editorial help from DC, DH, and YP.

### Conflict of interest statement

The authors declare that the research was conducted in the absence of any commercial or financial relationships that could be construed as a potential conflict of interest.

## Supplementary material

Supplementary Figure 1 Microbiota dysbiosis does not alter immune cell populations in the lungs. **(A)** The total numbers of F4/80^+^CD11c^+^ macrophages, CD11c^+^MHCII^+^ dendritic cells, CD11b^+^GR1^hi^ neutrophils, CD3^−^NK1.1^+^ NK cells, CD3^+^CD4^+^ T lymphocytes and CD19^+^ B cells were quantified by flow cytometry in lung homogenates from uninfected ABX vs. non-ABX mice. **(B)** Cytometry analysis of percentage of TNFα-producing F4/80^+^CD11c^+^ macrophages, TNFα-producing CD3^+^CD4^+^ T lymphocytes and IL-17A-producing CD3^+^CD4^+^ T lymphocytes in lungs from uninfected ABX vs. non-ABX mice. Data from 1–3 independent experiments (*n* = 4–5 mice/group/experiment) were pooled and the graphs show median with interquartile range of the pooled data (B cells, TNFα^+^ macrophages, TNFα^+^ CD4 T cells, IL-17A^+^ CD4 T cells) or mean ± SD (macrophages, dendritic cells, neutrophils, NK cells, CD4 T cells). Data were analyzed using the Mann-Whitney test (B cells, TNFα^+^ macrophages, TNFα^+^ CD4 T cells, IL-17A^+^ CD4 T cells) or using the unpaired Student's *t*-test (macrophages, dendritic cells, neutrophils, NK cells, CD4 T cells).

Supplementary Figure 2 Microbiota dysbiosis does not impact early inflammatory response to *M. tuberculosis* infection in lungs. **(A)** The expression of inflammatory cytokines and the antimicrobial peptide CRAMP was measured by RT-qPCR in the lungs of non-ABX vs. ABX mice 7 days p.i., Gene expression represents relative Ct value compared to *Hprt* Ct value (ΔCt) and the mean of ΔCt values in the control group (ΔΔCt). **(B)** The production of TNFα, IFNγ, and IL-12(p70) was measured in lung homogenates from control (non-ABX) and ABX mice 7 days p.i., Data from 2–3 independent experiments (*n* = 2–3 mice/group/experiment) were pooled and the graphs show mean ± SD (TNFα, TGFβ, CRAMP in **A**) or median with interquartile range (IL-1β, IL-6, IFNγ, IL-17A in **A**; TNFα, IFNγ, IL-12 in **B**) of the pooled data. Data were analyzed using the Student's *t*-test (TNFα, TGFβ, CRAMP in **A**) or the Mann-Whitney test (IL-1β, IL-6, IFNγ, IL-17A in **A**; TNFα, IFNγ, IL-12 in **B**).

Supplementary Figure 3 Microbiota dysbiosis does not alter innate myeloid and lymphoid cell populations during early lung colonization by *M. tuberculosis*. The total number of F4/80^+^CD11c^+^ macrophages, CD11c^+^MHCII^+^ dendritic cells, CD11b^+^GR1^hi^ neutrophils, and CD3^−^NK1.1^+^ NK cells were quantified by flow-cytometry in lung homogenates from *M. tuberculosis*-infected ABX vs. non-ABX mice 7 days p.i., Data from 4 independent experiments (*n* = 6–8 mice/group/experiment) were pooled and the graphs show median with interquartile range of the pooled data (macrophages, neutrophils) or mean ± SD (dendritic cells, NK cells). Data were analyzed using the Mann-Whitney test (macrophages, neutrophils) or using the unpaired Student's *t*-test (dendritic cells, NK cells).

Supplementary Figure 4 NKT and γ/δ T cells are not modified in infected microbiota-altered mice. **(A)** Gating strategy to analyze unconventional innate-like lymphocytes, namely NKT cells (CD3^+^NK1.1^+^) and γ/δ T cells (CD3^+^TCRγ/δ^+^) by flow cytometry. **(B,C) (B)** Total NKT cells (left) and γ/δ T cells (right) and **(C)** percentages in the lungs of control (non-ABX) and ABX mice 7 days p.i., Data from 2 independent experiments (*n* = 8 mice/group/experiment) were pooled and the graphs show the mean ± SD of the pooled data. Data were analyzed using the unpaired Student's *t*-test.

Supplementary Table 1 Primers used in the study for RT-qPCR.

Supplementary Table 2 Antibodies used in the study for flow cytometry.
