# Supplementary figures and images for: The Host Microbiota Contributes to Early Protection Against Lung Colonization by Mycobacterium tuberculosis
Source: Front Immunol. 2018 Nov 14;9:2656. doi: 10.3389/fimmu.2018.02656 (PMC6246741; doi:10.3389/fimmu.2018.02656)

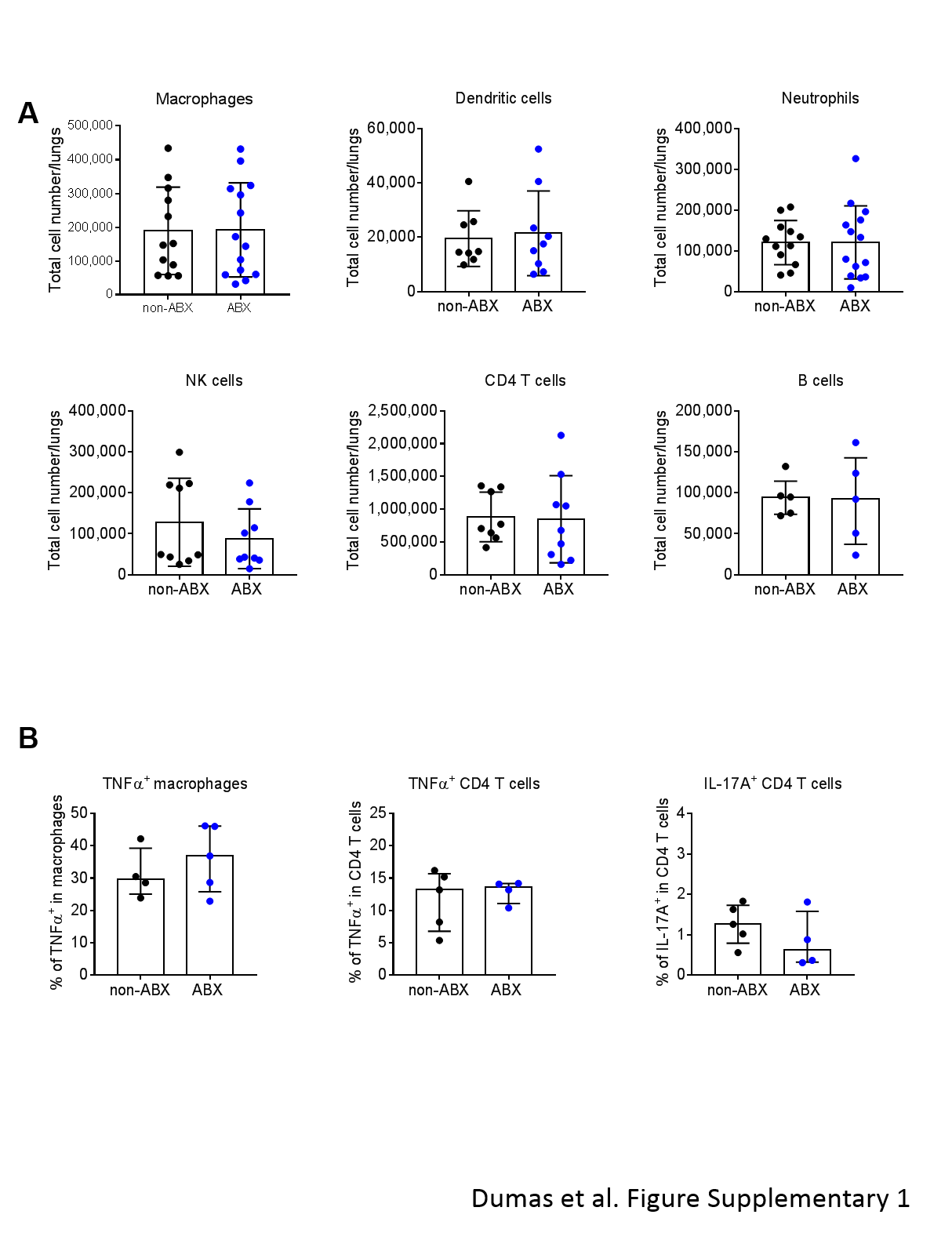

Supplement: Supplementary Figure 1 — Microbiota dysbiosis does not alter immune cell populations in the lungs. (A) The total numbers of F4/80+CD11c+ macrophages, CD11c+MHCII+ dendritic cells, CD11b+GR1hi neutrophils, CD3−NK1.1+ NK cells, CD3+CD4+ T lymphocytes and CD19+ B cells were quantified by flow cytometry in lung homogenates from uninfected ABX vs. non-ABX mice. (B) Cytometry analysis of percentage of TNFα-producing F4/80+CD11c+ macrophages, TNFα-producing CD3+CD4+ T lymphocytes and IL-17A-producing CD3+CD4+ T lymphocytes in lungs from uninfected ABX vs. non-ABX mice. Data from 1–3 independent experiments (n = 4–5 mice/group/experiment) were pooled and the graphs show median with interquartile range of the pooled data (B cells, TNFα+ macrophages, TNFα+ CD4 T cells, IL-17A+ CD4 T cells) or mean ± SD (macrophages, dendritic cells, neutrophils, NK cells, CD4 T cells). Data were analyzed using the Mann-Whitney test (B cells, TNFα+ macrophages, TNFα+ CD4 T cells, IL-17A+ CD4 T cells) or using the unpaired Student's t-test (macrophages, dendritic cells, neutrophils, NK cells, CD4 T cells). [file Image_1.TIF]

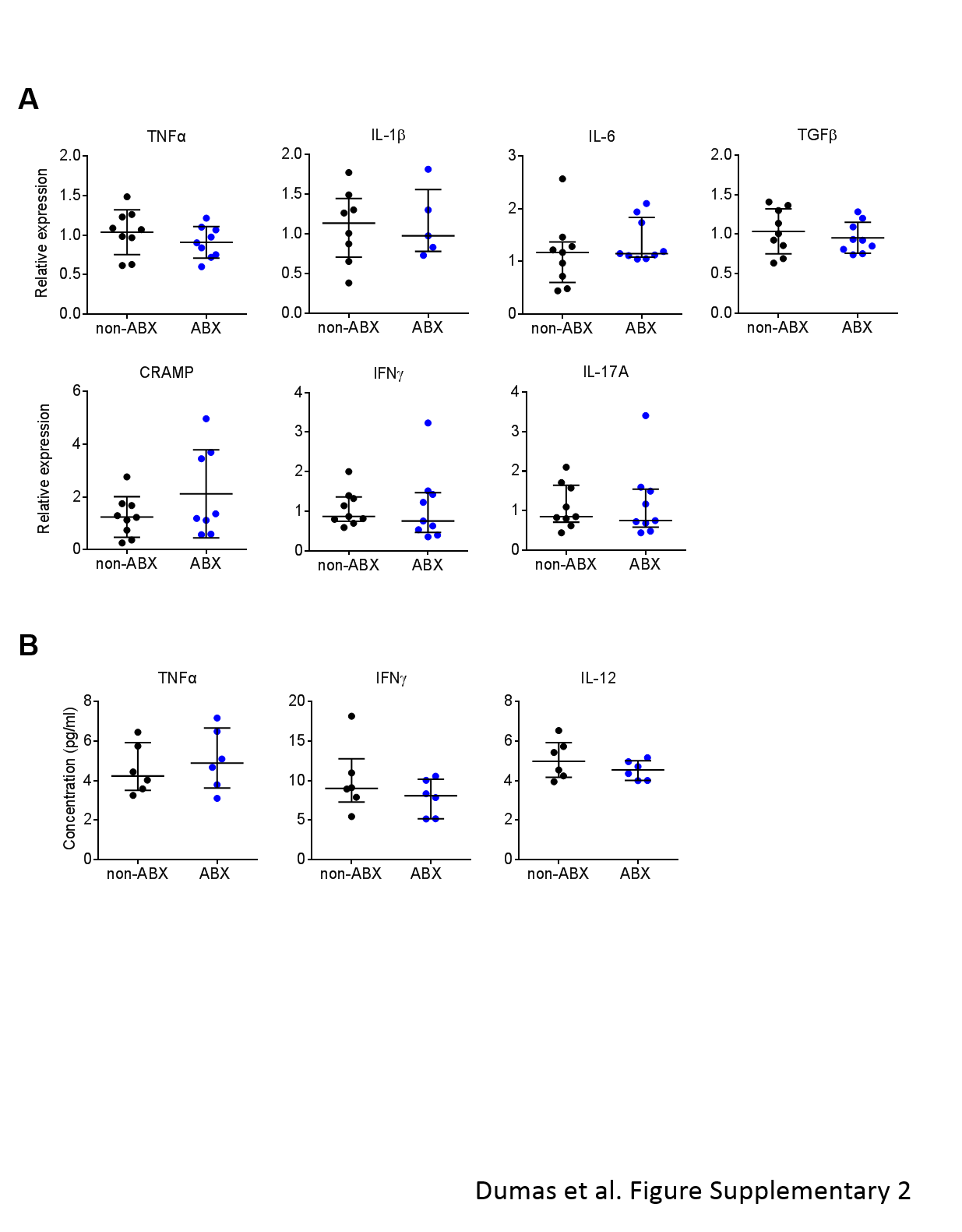

Supplement: Supplementary Figure 2 — Microbiota dysbiosis does not impact early inflammatory response to M. tuberculosis infection in lungs. (A) The expression of inflammatory cytokines and the antimicrobial peptide CRAMP was measured by RT-qPCR in the lungs of non-ABX vs. ABX mice 7 days p.i., Gene expression represents relative Ct value compared to Hprt Ct value (ΔCt) and the mean of ΔCt values in the control group (ΔΔCt). (B) The production of TNFα, IFNγ, and IL-12(p70) was measured in lung homogenates from control (non-ABX) and ABX mice 7 days p.i., Data from 2–3 independent experiments (n = 2–3 mice/group/experiment) were pooled and the graphs show mean ± SD (TNFα, TGFβ, CRAMP in A) or median with interquartile range (IL-1β, IL-6, IFNγ, IL-17A in A; TNFα, IFNγ, IL-12 in B) of the pooled data. Data were analyzed using the Student's t-test (TNFα, TGFβ, CRAMP in A) or the Mann-Whitney test (IL-1β, IL-6, IFNγ, IL-17A in A; TNFα, IFNγ, IL-12 in B). [file Image_2.TIF]

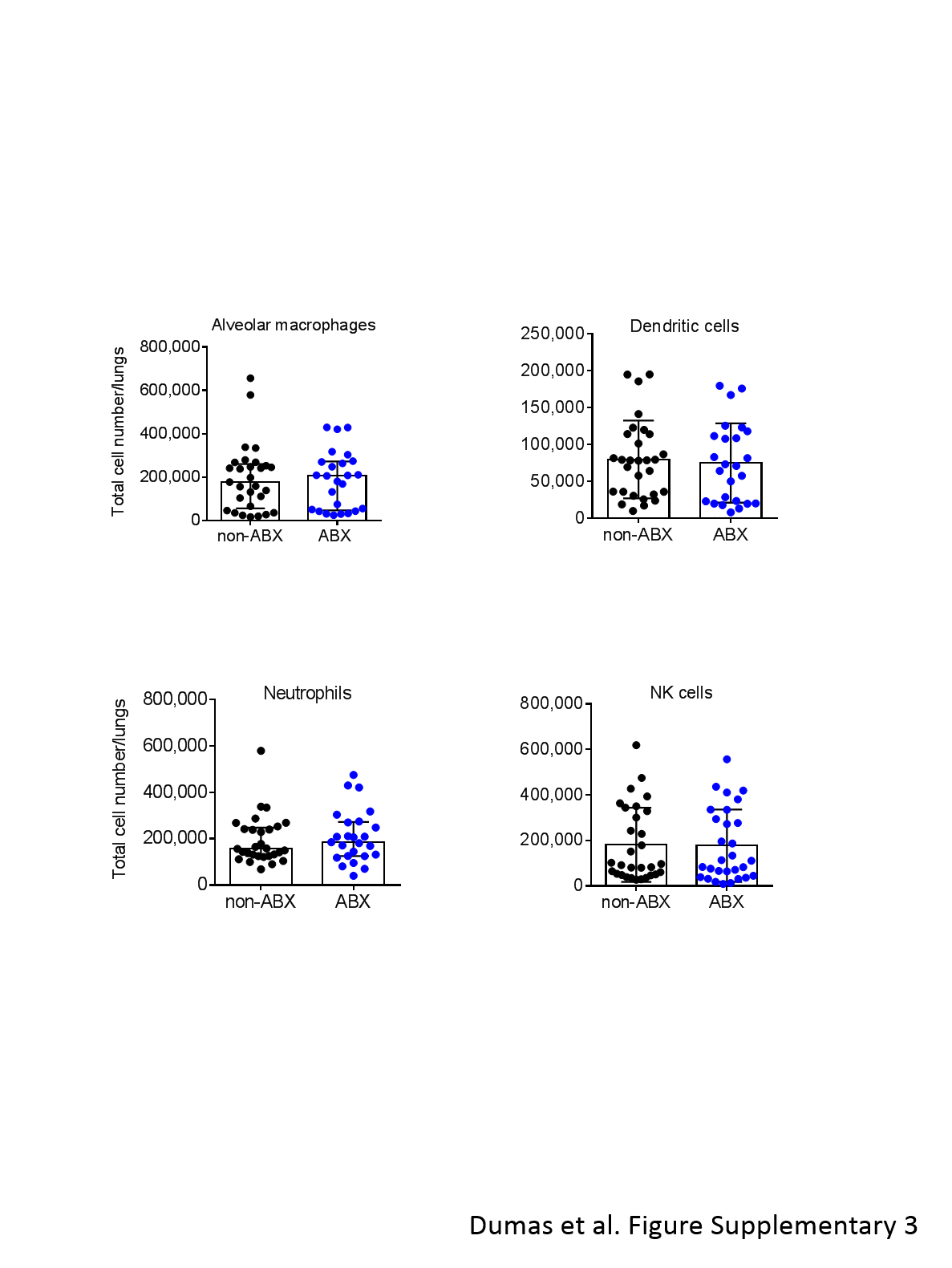

Supplement: Supplementary Figure 3 — Microbiota dysbiosis does not alter innate myeloid and lymphoid cell populations during early lung colonization by M. tuberculosis. The total number of F4/80+CD11c+ macrophages, CD11c+MHCII+ dendritic cells, CD11b+GR1hi neutrophils, and CD3−NK1.1+ NK cells were quantified by flow-cytometry in lung homogenates from M. tuberculosis-infected ABX vs. non-ABX mice 7 days p.i., Data from 4 independent experiments (n = 6–8 mice/group/experiment) were pooled and the graphs show median with interquartile range of the pooled data (macrophages, neutrophils) or mean ± SD (dendritic cells, NK cells). Data were analyzed using the Mann-Whitney test (macrophages, neutrophils) or using the unpaired Student's t-test (dendritic cells, NK cells). [file Image_3.TIF]

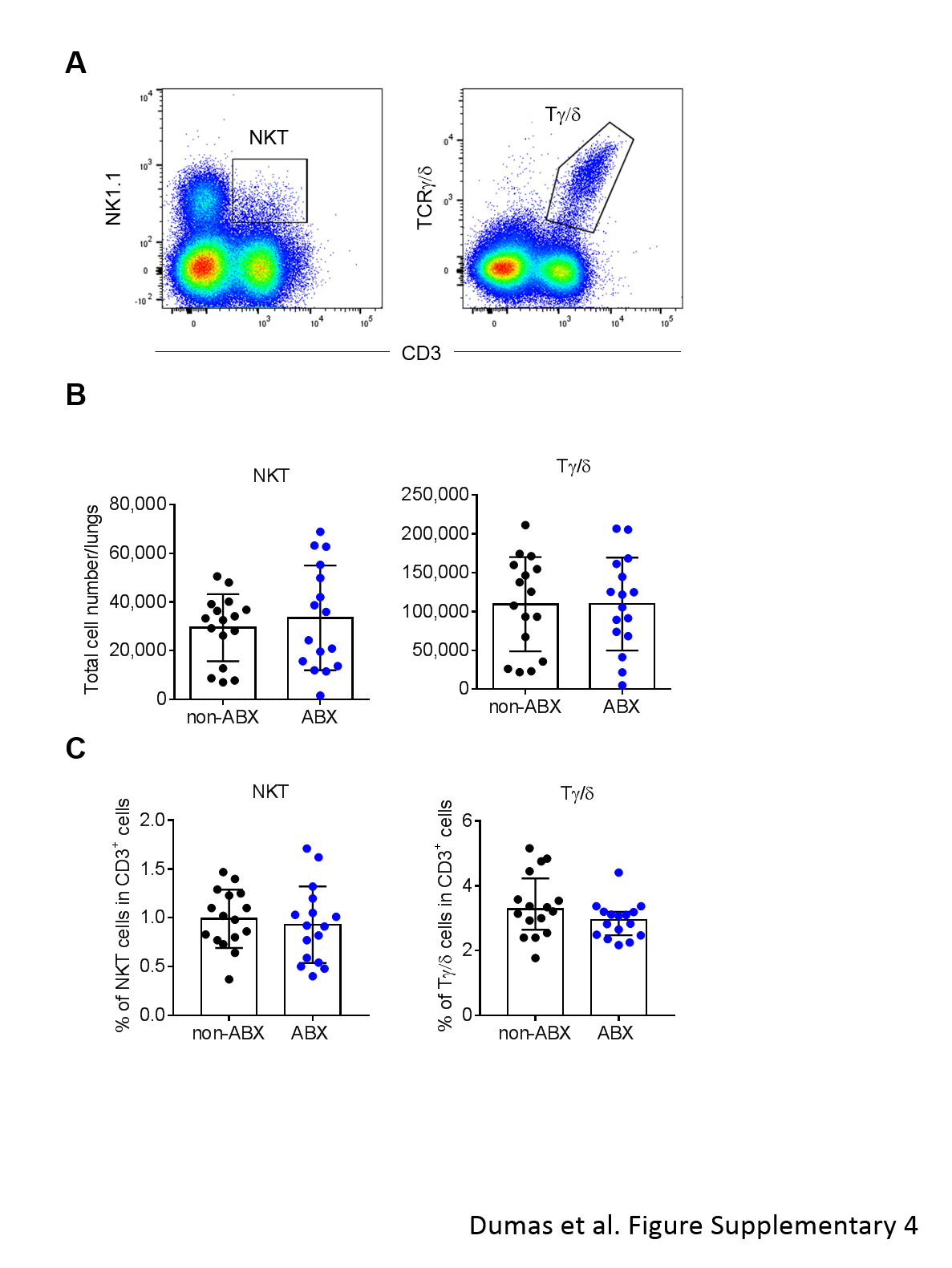

Supplement: Supplementary Figure 4 — NKT and γ/δ T cells are not modified in infected microbiota-altered mice. (A) Gating strategy to analyze unconventional innate-like lymphocytes, namely NKT cells (CD3+NK1.1+) and γ/δ T cells (CD3+TCRγ/δ+) by flow cytometry. (B,C) (B) Total NKT cells (left) and γ/δ T cells (right) and (C) percentages in the lungs of control (non-ABX) and ABX mice 7 days p.i., Data from 2 independent experiments (n = 8 mice/group/experiment) were pooled and the graphs show the mean ± SD of the pooled data. Data were analyzed using the unpaired Student's t-test. [file Image_4.TIF]
